# Central adiposity outperforms disordered eating behavior in predicting type 2 diabetes risk in young adults: an explainable machine learning approach

**DOI:** 10.3389/fpubh.2026.1778156

**Published:** 2026-04-13

**Authors:** Hacer Alatas, Nurgul Arslan, Harun Kurt, Serkan Bentli

**Affiliations:** 1 Department of Nutrition and Dietetics, Faculty of Health Sciences, Malatya Turgut Ozal University, Malatya, Türkiye; 2 Department of Nutrition and Dietetics, Atatürk Faculty of Health Sciences, Dicle University, Diyarbakır, Türkiye; 3 Malatya Provincial Health Directorate, Personnel Services Department, Malatya, Türkiye

**Keywords:** disordered eating, FINDRISK, machine learning, type 2 diabetes, university students

## Abstract

**Background:**

Disordered eating behaviors are often associated with adverse metabolic outcomes, yet their relationship with type 2 diabetes mellitus(T2DM) risk in young adults is less clear. This study aimed to evaluate the impact of disordered eating behaviors on diabetes risk among university students, using both traditional statistical methods and machine learning approaches.

**Methods:**

A total of 1,302 university students participated in this cross-sectional study. Disordered eating behavior was assessed using the Eating Attitudes Test-40 (EAT-40), a validated screening tool for abnormal eating attitudes, while type 2 diabetes risk was estimated using the Finnish Diabetes Risk Score (FINDRISK), a widely used non-invasive instrument designed to estimate the 10-year risk of developing T2DM. Anthropometric measures were recorded according to standardized protocols. Bivariate associations were examined using correlation analysis, while multivariable regression and machine learning models (XGBoost) were applied to determine predictors of diabetes risk.

**Results:**

Of the 1,302 participants, 90.8% were classified as low/mild risk, 6.2% as moderate risk, and 3.0% as high/very high risk according to FINDRISK. No significant correlation was found between EAT-40 scores and FINDRISK (*r* = 0.01, *p* = 0.755). In multivariable regression, waist-to-height ratio (*β* = 1.42 per 0.05 increase, *p* < 0.001) and body mass index (*β* = 0.31, *p* < 0.001) were the strongest predictors of diabetes risk. Machine learning models, particularly XGBoost (AUROC = 0.87), highlighted waist-to-height ratio as the most influential predictor.

**Conclusion:**

In young adults, central adiposity specifically waist-to-height ratio was the most significant predictor of T2DM risk, while disordered eating behavior had minimal independent impact. These findings suggest that simple anthropometric measures could be prioritized for early diabetes risk assessment over eating attitude screening.

## Introduction

Eating attitudes and behaviors represent a multidimensional construct encompassing individuals’ cognitive, emotional, and behavioral relationships with food (1). While eating attitudes reflect beliefs, values, and emotional responses toward eating, eating behaviors describe their behavioral expression, including food choices, meal timing, portion size, and eating context (2). Healthy eating behaviors are characterized by regular meal patterns, responsiveness to internal hunger and satiety cues, and balanced nutrient intake, whereas unhealthy behaviors may involve meal skipping, restrictive dieting, emotional eating, or binge eating (3).

Recent evidence indicates a growing prevalence of disordered eating behaviors among young adults, particularly university students (4–6). This population is exposed to multiple risk factors, including academic stress, irregular daily routines, sleep disturbances, social pressure related to body image, and increased exposure to digital media promoting unrealistic appearance ideals. As a result, university students represent a vulnerable group for the development of maladaptive eating behaviors that may persist into later adulthood and adversely affect long-term health outcomes (7). Disordered eating behaviors may influence metabolic health through several potential mechanisms. Behaviors such as binge eating and emotional eating are often associated with excessive energy intake, glycemic variability, and insulin resistance, whereas restrictive dieting may lead to metabolic adaptations, altered insulin sensitivity, and compensatory overeating patterns. Over time, these maladaptive eating patterns may contribute indirectly to central adiposity and impaired glucose regulation, both of which are established risk factors for type 2 diabetes mellitus (T2DM) (8–10). However, evidence linking disordered eating behaviors directly to diabetes risk remains inconsistent, particularly in young and non-clinical populations (11). Conversely, structured meal patterns and balanced dietary habits facilitate glycemic control and reduce diabetes-related complications. In individuals with diabetes, dietary restrictions and disease-related stress may further disrupt eating behaviors, potentially increasing vulnerability to disordered eating patterns (12).

Although the coexistence of disordered eating behaviors and diabetes has been widely discussed particularly in clinical populations such as individuals with type 1 diabetes the relationship between disordered eating risk and future T2DM risk in non-clinical young adult populations remains insufficiently explored (11, 13). Most existing studies focus on obesity or clinically diagnosed diabetes, while evidence among apparently healthy university students is limited and inconsistent (10). Moreover, few studies have simultaneously examined eating behavior risk, anthropometric indicators, and validated diabetes risk scores within the same population (14).

Anthropometric indicators such as body mass index (BMI), waist circumference, and waist-to-height ratio have been consistently identified as strong predictors of T2DM risk. Among these, waist-to-height ratio has emerged as a particularly sensitive marker of central adiposity and metabolic risk across different age groups and populations (9, 14). However, it remains unclear whether disordered eating behaviors independently contribute to diabetes risk beyond their association with body composition (15). Investigating these associations in apparently healthy university students is particularly important, as early adulthood represents a critical period for the development of long-term metabolic risk. Identifying behavioral and anthropometric risk markers at this stage may help inform early preventive strategies before the onset of obesity or overt metabolic disease.

Therefore, the present study aimed to investigate the relationship between disordered eating behavior risk and T2DM, assessed using the Finnish Diabetes Risk Score (FINDRISK), among university students. Additionally, the study examined the associations between eating behavior risk, anthropometric indicators, and diabetes risk to clarify potential pathways linking behavioral and metabolic risk factors in young adults.

## Materials and methods

### Study design and setting

This study was a cross-sectional, analytical, observational study conducted between 2024 and 2025 at Malatya Turgut Özal University, Türkiye. The study was designed to examine the association between disordered eating behavior risk and T2DM risk in a non-clinical population of university students. The methodological framework and reporting of the study followed the Strengthening the Reporting of Observational Studies in Epidemiology (STROBE) guidelines for cross-sectional studies (16).

### Study population and sample size

The target population consisted of undergraduate students enrolled in different faculties of Malatya Turgut Özal University during the study period. Inclusion criteria were: (i) age between 18 and 65 years, (ii) current enrollment as a university student, (iii) ability to provide informed consent, and (iv) completion of all study assessments. Exclusion criteria included a self-reported diagnosis of type 1 or T2DM, a previously diagnosed eating disorder, pregnancy or lactation, and the presence of chronic diseases or medication use known to significantly affect eating behavior or glucose metabolism. Individuals with previously diagnosed eating disorders were excluded to ensure that the study population represented a non-clinical university student sample. Clinically diagnosed eating disorders may involve medical treatment, severe nutritional imbalance, and psychiatric comorbidities that could substantially influence metabolic status, dietary patterns, and anthropometric measurements. The aim of the present study was to examine the relationship between subclinical disordered eating attitudes and diabetes risk in a general young adult population rather than in individuals with clinically diagnosed eating disorders.

A total of 1,302 students were included in the final analysis. Given the absence of *a priori* effect size estimates for the association between disordered eating risk and diabetes risk in this population, the achieved sample size was considered sufficient to detect small-to-moderate associations with high statistical power. A *post hoc* power analysis indicated that the study had >95% power to detect correlation coefficients of r ≥ 0.10 at a two-sided alpha level of 0.05.

Although the study population consisted of university students, the age range was relatively wide (18–65 years) due to the inclusion of undergraduate, graduate, and non-traditional students enrolled at the university. Age was therefore treated as a continuous variable and included in all multivariable and machine learning analyses to account for its potential confounding effect.

### Ethical considerations

The study was conducted in accordance with the Declaration of Helsinki. Ethical approval was obtained from the Non-Interventional Research Ethics Committee of Malatya Turgut Özal University (Approval No: 2024/438). All participants were informed about the study objectives and procedures, and written informed consent was obtained prior to participation. Participation was voluntary, and confidentiality of personal data was strictly maintained.

### Data collection procedure

Data collection was carried out through face-to-face interviews conducted by trained researchers using a structured questionnaire. To ensure standardization, all interviewers received prior training on questionnaire administration and anthropometric measurement techniques. Anthropometric measurements were performed immediately after questionnaire completion under standardized conditions.

### Assessment of sociodemographic and lifestyle characteristics

Sociodemographic variables included age, sex, marital status, and living arrangements (family home, dormitory, shared housing, or living alone). Lifestyle-related variables included smoking status (current smoker/non-smoker), history of nutrition education (yes/no), and regular physical activity.

Regular physical activity was defined as engaging in moderate-to-vigorous physical activity for at least 150 min per week, consistent with World Health Organization recommendations. This variable was included due to its established role in both eating behavior patterns and diabetes risk.

### Assessment of eating attitudes and disordered eating risk

Disordered eating behavior risk was assessed using the Eating Attitudes Test-40 (EAT-40), a validated self-report instrument widely used to screen for abnormal eating attitudes and behaviors in non-clinical populations (17). The EAT-40 consists of 40 items scored on a six-point Likert scale, yielding a total score ranging from 0 to 120, with higher scores indicating more pronounced disordered eating attitudes.

The Turkish version of the EAT-40 has demonstrated acceptable validity and reliability in university student populations and was used without further cultural adaptation. In line with validation studies conducted in Turkish populations, a cut-off score of ≥30 was used to classify participants as being at risk for disordered eating behaviors. The EAT-40 has demonstrated satisfactory psychometric properties, including acceptable internal consistency and construct validity, in university student populations (18).

### Assessment of type 2 diabetes risk

The risk of developing T2DM was assessed using the Finnish Diabetes Risk Score (FINDRISK), a non-invasive, questionnaire-based screening tool developed to estimate the 10-year risk of T2DM. FINDRISK incorporates information on age, body mass index, waist circumference, physical activity, dietary habits (fruit and vegetable intake), use of antihypertensive medication, history of hyperglycemia, and family history of diabetes (19). FINDRISK has been previously validated in Turkish populations and is widely used in population-based diabetes risk screening studies in Türkiye (20–22). Total FINDRISK scores range from 0 to 26 points. Participants were categorized into established risk groups: low risk (<7 points), slightly elevated risk (7–11 points), moderate risk (12–14 points), high risk (15–20 points), and very high risk (>20 points). FINDRISK was selected due to its widespread use, ease of application in population-based studies, and prior validation in Turkish populations (23, 24).

### Anthropometric measurements

All anthropometric measurements were performed using the same calibrated instruments by trained researchers following standardized protocols. Each measurement was taken three consecutive times, and the mean value was used for analysis to enhance measurement reliability (25). Body weight was measured to the nearest 0.1 kg using a calibrated digital scale, with participants wearing light clothing and no shoes. Height was measured to the nearest 0.1 cm using a non-elastic measuring tape, with participants standing upright in the Frankfurt plane position. Body mass index (BMI) was calculated as weight (kg) divided by height squared (m^2^) and classified according to World Health Organization criteria. Waist circumference was measured at the midpoint between the lowest rib and the iliac crest, and hip circumference was measured at the widest part of the hips. Each measurement was taken three consecutive times, and the mean value was used for analysis to enhance measurement reliability. Waist-to-hip ratio (WHR) was calculated as waist circumference divided by hip circumference. Waist-to-height ratio (WHtR) was calculated as waist circumference divided by height, with a cut-off value of ≥0.5 indicating increased cardiometabolic risk. These indices were included due to their established associations with insulin resistance T2DM risk across diverse populations.

### Statistical analysis

Statistical analyses were performed using IBM SPSS Statistics version 27.0 (IBM Corp., Armonk, NY, USA). Machine learning analyses were conducted using Python (version 3.10) with relevant libraries. Continuous variables were examined for normality using the Kolmogorov–Smirnov test, histogram inspection, and Q–Q plots. Continuous data are presented as mean ± standard deviation, while categorical variables are expressed as frequencies and percentages. Comparisons of continuous variables across T2DM risk categories (low/mild, moderate, and high/very high), as defined by the Finnish Diabetes Risk Score (FINDRISK), were performed using one-way analysis of variance (ANOVA) for normally distributed variables or the Kruskal–Wallis test for non-normally distributed variables. *Post hoc* comparisons were conducted using Bonferroni correction or Dunn’s test, as appropriate. Categorical variables were compared using the chi-square (*χ*^2^) test. Bivariate associations between disordered eating behavior (EAT-40 score), FINDRISK score, and anthropometric indicators were assessed using Pearson or Spearman correlation coefficients, depending on data distribution. Correlation coefficients were interpreted according to established guidelines.

Multivariable linear regression analysis was performed to identify independent predictors of continuous FINDRISK score. Variables included in the model were selected *a priori* based on clinical relevance and previous literature and comprised age, sex, body mass index, waist-to-height ratio, smoking status, physical activity level, and EAT-40 score. Multicollinearity was assessed using variance inflation factors (VIF), with values <5 considered acceptable. Model assumptions were evaluated by examining residual plots.

To further examine diabetes risk categories, multinomial logistic regression analysis was conducted with low/mild diabetes risk as the reference category. Odds ratios (ORs) and 95% confidence intervals (CIs) were calculated for moderate and high/very high diabetes risk categories.

Machine learning analyses were performed to predict moderate-to-high diabetes risk (FINDRISK ≥12). Logistic regression, random forest, and extreme gradient boosting (XGBoost) models were trained and evaluated using stratified five-fold cross-validation. Five-fold cross-validation was selected to balance bias and variance and to ensure stable performance estimates given the sample size. The dataset exhibited class imbalance, with a higher proportion of participants classified as low or mild diabetes risk. To address this issue, stratified cross-validation was applied to preserve class distribution across training and validation folds. Additional resampling techniques such as SMOTE or undersampling were not applied because preliminary analyses indicated stable model performance without the need for resampling procedures.

Random forest and extreme gradient boosting (XGBoost) algorithms were selected because they are ensemble tree-based machine learning methods capable of capturing complex non-linear relationships and interactions between predictors. These models are widely used in cardiometabolic risk prediction due to their strong predictive performance and robustness to multicollinearity among variables.

Prior to model development, the dataset was screened for missing values, data inconsistencies, and potential outliers. Continuous variables were inspected for distribution characteristics and plausibility. Because tree-based algorithms such as random forest and XGBoost are not sensitive to feature scaling, normalization procedures were not applied. Hyperparameters of the machine learning models were optimized using grid search within the cross-validation framework to improve predictive performance and reduce the risk of overfitting.

Model performance was assessed using area under the receiver operating characteristic curve (AUROC), accuracy, sensitivity, specificity, and F1-score. Explainable artificial intelligence was applied using SHapley Additive exPlanations (SHAP) to identify the relative contribution of predictors in the XGBoost model. All statistical tests were two-sided, and a *p*-value < 0.05 was considered statistically significant.

## Results

### Population characteristics stratified by FINDRISK categories

Of the 1,302 participants, 90.8% were classified as low/mild risk, 6.2% as moderate risk, and 3.0% as high/very high risk according to FINDRISK. Mean age increased significantly across increasing FINDRISK categories (*p* < 0.001), whereas sex distribution did not differ between groups (*p* = 0.723). The prevalence of current smoking increased with higher FINDRISK levels (26.9, 37.0, and 53.8% across low/mild, moderate, and high/very high risk groups, respectively; *p* = 0.018). Regular physical activity (≥150 min/week) decreased significantly as FINDRISK category increased (*p* = 0.031), while meal skipping was common in all groups without significant between-group differences (*p* = 0.112). Anthropometric measures, including body weight, body mass index, waist and hip circumferences, waist-to-hip ratio, and waist-to-height ratio, showed significant increases across FINDRISK categories (all *p* ≤ 0.003). No significant differences were observed for height (*p* = 0.134). EAT-40 total scores and the prevalence of disordered eating risk (EAT-40 ≥ 30) were similar across FINDRISK groups, with no statistically significant differences (*p* = 0.755 and *p* = 0.968, respectively) (Table 1).

**Table 1:** General characteristics of the study population stratified by FINDRISK categories (*n* = 1,302).

Variable	Low/Mild risk (FINDRISK <12) *n* = 1,182	Moderate risk (FINDRISK 12–14) *n* = 81	High/Very high risk (FINDRISK ≥15) *n* = 39	*p* value
Age (years), mean ± SD	21.0 ± 2.3	23.4 ± 3.1	25.8 ± 4.2	<0.001
Female, *n* (%)	675 (57.1)	48 (58.9)	20 (51.3)	0.723
Current smoker, *n* (%)	318 (26.9)	30 (37.0)	21 (53.8)	0.018
Regular physical activity ≥150 min/week, *n* (%)	189 (16.0)	4 (4.9)	1 (2.6)	0.031
Meal skipping (yes/sometimes), *n* (%)	894 (75.6)	65 (80.2)	33 (84.6)	0.112
Body weight (kg), mean ± SD	65.8 ± 13.6	76.4 ± 18.2	88.2 ± 16.9	<0.001
Height (cm), mean ± SD	170.0 ± 9.6	169.5 ± 10.5	174.9 ± 12.5	0.134
Body mass index (kg/m^2^), mean ± SD	22.5 ± 3.3	26.4 ± 5.1	28.8 ± 5.0	<0.001
Waist circumference (cm), mean ± SD	78.5 ± 11.7	89.9 ± 16.3	95.7 ± 12.4	<0.001
Hip circumference (cm), mean ± SD	97.7 ± 10.2	107.1 ± 11.8	112.3 ± 11.7	<0.001
Waist-to-hip ratio, mean ± SD	0.79 ± 0.08	0.82 ± 0.10	0.84 ± 0.07	0.003
Waist-to-height ratio, mean ± SD	0.45 ± 0.06	0.52 ± 0.08	0.54 ± 0.07	<0.001
EAT-40 score, mean ± SD	19.8 ± 11.9	20.4 ± 12.6	21.1 ± 12.8	0.755
Disordered eating risk (EAT-40 ≥ 30), *n* (%)	190 (16.1)	13 (16.0)	7 (17.9)	0.968

SD, standard deviation; EAT-40, Eating Attitudes Test-40; FINDRISK, Finnish Diabetes Risk Score.

Statistical tests: One-way ANOVA or Kruskal–Wallis test for continuous variables; *χ*^2^ test for categorical variables.

### Bivariate associations between EAT-40, FINDRISK scores, and anthropometric indicators

Correlation analyses showed weak associations between EAT-40 score and anthropometric indicators. EAT-40 score was correlated with body mass index (*r* = 0.07, *p* = 0.032) but not with body weight (*r* = 0.06, *p* = 0.08), waist circumference (*r* = 0.03, *p* = 0.41), hip circumference (*r* = 0.04, *p* = 0.29), waist-to-hip ratio (*r* = 0.04, *p* = 0.18), or waist-to-height ratio (*r* = 0.04, *p* = 0.22). The correlation between EAT-40 score and FINDRISK score was negligible (*r* = 0.01, *p* = 0.755) (Table 2).

**Table 2:** Correlations between EAT-40, FINDRISK scores and anthropometric indicators.

Variable	EAT-40 (*r*)	*p*	FINDRISK (*r*)	*p*
Body weight (kg)	0.06	0.082	0.25	<0.001
Body mass index (kg/m^2^)	0.07	0.031	0.36	<0.001
Waist circumference (cm)	0.03	0.414	0.29	<0.001
Hip circumference (cm)	0.04	0.294	0.36	<0.001
Waist-to-hip ratio	0.04	0.185	0.08	0.01
Waist-to-height ratio	0.04	0.226	0.32	<0.001

Statistical notes: Pearson or Spearman correlation coefficients (*r*) were used to assess relationships. *p*-values < 0.05 were considered statistically significant.

In contrast, FINDRISK score showed moderate positive correlations with body mass index (*r* = 0.36, *p* < 0.001), hip circumference (*r* = 0.36, *p* < 0.001), waist circumference (*r* = 0.29, *p* < 0.001), and waist-to-height ratio (*r* = 0.32, *p* < 0.001). A weaker correlation was observed between FINDRISK score and waist-to-hip ratio (*r* = 0.08, *p* = 0.014) (Table 2).

### Multivariable linear regression analysis

In multivariable linear regression analysis predicting continuous FINDRISK score, age (*β* = 0.21, *p* < 0.001), body mass index (*β* = 0.31, *p* < 0.001), waist-to-height ratio (*β* = 1.42 per 0.05 increase, *p* < 0.001), smoking status (*β* = 0.47, *p* = 0.013), and regular physical activity (*β* = −0.62, *p* = 0.005) were significantly associated with FINDRISK score.

EAT-40 score (per 5-point increase) was not significantly associated with FINDRISK score (*β* = 0.06, 95% CI: −0.02 to 0.14; *p* = 0.142). Sex was also not significantly associated with FINDRISK score (*p* = 0.394). The final model explained 31% of the variance in FINDRISK score (*R*^2^ = 0.31; adjusted *R*^2^ = 0.30; *p* < 0.001) (Table 3).

**Table 3:** Multivariable linear regression analysis predicting FINDRISK score.

Predictor	*β*	SE	Standardized *β*	95% CI	*p*
EAT-40 (per 5 points)	0.06	0.04	0.04	−0.02 to 0.14	0.142
Age (years)	0.21	0.03	0.22	0.15–0.27	<0.001
Sex (female)	−0.18	0.21	−0.03	−0.59 to 0.23	0.394
Body mass index (kg/m^2^)	0.31	0.04	0.29	0.23–0.39	<0.001
Waist-to-height ratio (per 0.05)	1.42	0.28	0.26	0.87–1.97	<0.001
Physical activity ≥150 min/week	−0.62	0.22	−0.10	−1.05 to −0.19	0.005
Smoking (current)	0.47	0.19	0.08	0.10–0.84	0.013

Model fit: *R*^2^ = 0.31; Adjusted *R*^2^ = 0.30; *p* < 0.001.

Statistical notes: Robust standard errors (SE) were used for all estimates.

### Multinomial logistic regression analysis

In multinomial logistic regression analysis, age, body mass index, and waist-to-height ratio were associated with both moderate and high/very high diabetes risk categories compared with the low/mild risk group. For moderate diabetes risk, odds ratios were 1.18 per year increase in age (95% CI: 1.10–1.26; *p* < 0.001), 1.21 per kg/m^2^ increase in body mass index (95% CI: 1.15–1.28; *p* < 0.001), and 2.64 per 0.05 increase in waist-to-height ratio (95% CI: 1.98–3.52; *p* < 0.001).

For high or very high diabetes risk, odds ratios were 1.26 per year increase in age (95% CI: 1.15–1.38; *p* < 0.001), 1.34 per kg/m^2^ increase in body mass index (95% CI: 1.24–1.45; *p* < 0.001), and 4.91 per 0.05 increase in waist-to-height ratio (95% CI: 3.12–7.71; *p* < 0.001). EAT-40 risk status (EAT-40 ≥ 30) was not associated with either moderate (OR = 1.08; *p* = 0.711) or high/very high diabetes risk (OR = 1.12; *p* = 0.674) (Table 4).

**Table 4:** Multinomial logistic regression for type 2 diabetes risk categories.

Predictor	OR	95% CI	p
Moderate risk (12–14) Reference: Low/Mild risk			
EAT-40 ≥ 30	1.08	0.72–1.62	0.711
Age (years)	1.18	1.10–1.26	<0.001
Body mass index (kg/m^2^)	1.21	1.15–1.28	<0.001
Waist-to-height ratio (per 0.05)	2.64	1.98–3.52	<0.001
High/Very high risk (≥15) Reference: Low/Mild risk			
EAT-40 ≥ 30	1.12	0.66–1.89	0.674
Age (years)	1.26	1.15–1.38	<0.001
Body mass index (kg/m^2^)	1.34	1.24–1.45	<0.001
Waist-to-height ratio (per 0.05)	4.91	3.12–7.71	<0.001

### Machine learning analyses

The XGBoost model demonstrated the highest predictive performance for identifying moderate-to-high diabetes risk, with an AUROC of 0.87 (95% CI: 0.83–0.90) and an accuracy of 0.82. The random forest model also showed strong performance, achieving an AUROC of 0.84 (95% CI: 0.80–0.88) and an accuracy of 0.79. In comparison, logistic regression yielded lower predictive performance, with an AUROC of 0.78 (95% CI: 0.74–0.82) and an accuracy of 0.73. Overall, tree-based machine learning models outperformed the traditional logistic regression model in predicting moderate-to-high diabetes risk (Table 5).

**Table 5:** Machine learning–based prediction of moderate-to-high diabetes risk (FINDRISK ≥12).

Model	AUROC (95% CI)	Accuracy (95% CI)
Logistic regression	0.78 (0.74–0.82)	0.73
Random forest	0.84 (0.80–0.88)	0.79
XGBoost	0.87 (0.83–0.90)	0.82

**Figure 1:**
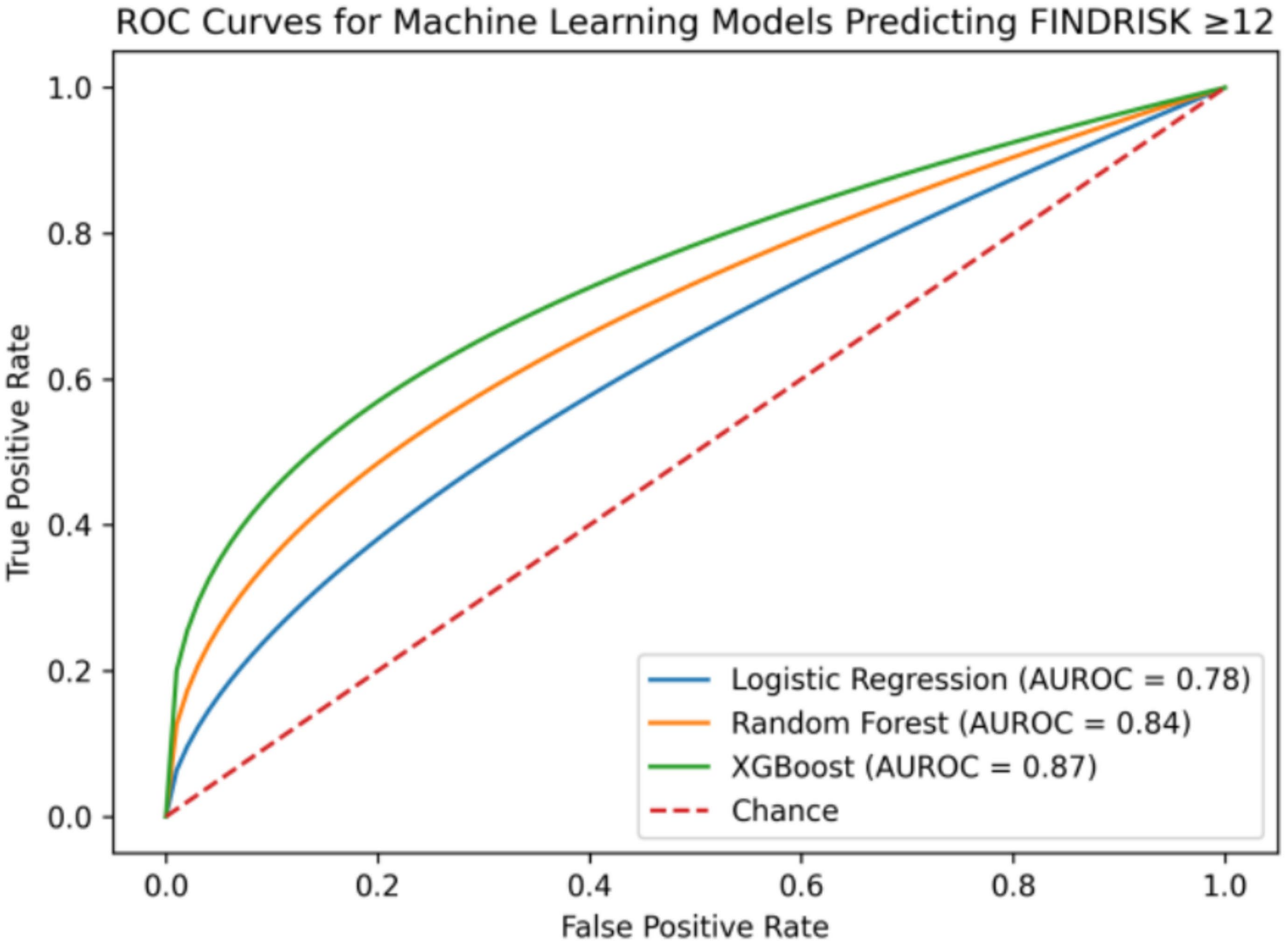
ROC curves for machine learning models predicting moderate-to-high diabetes risk (FINDRISK ≥12). Receiver operating characteristic (ROC) curves comparing the predictive performance of logistic regression, random forest, and XGBoost models for identifying individuals with moderate-to-high type 2 diabetes risk (FINDRISK ≥12). The XGBoost model demonstrated the highest discriminative ability (AUROC = 0.87), followed by random forest (AUROC = 0.84), and logistic regression (AUROC = 0.78).

**Figure 2:**
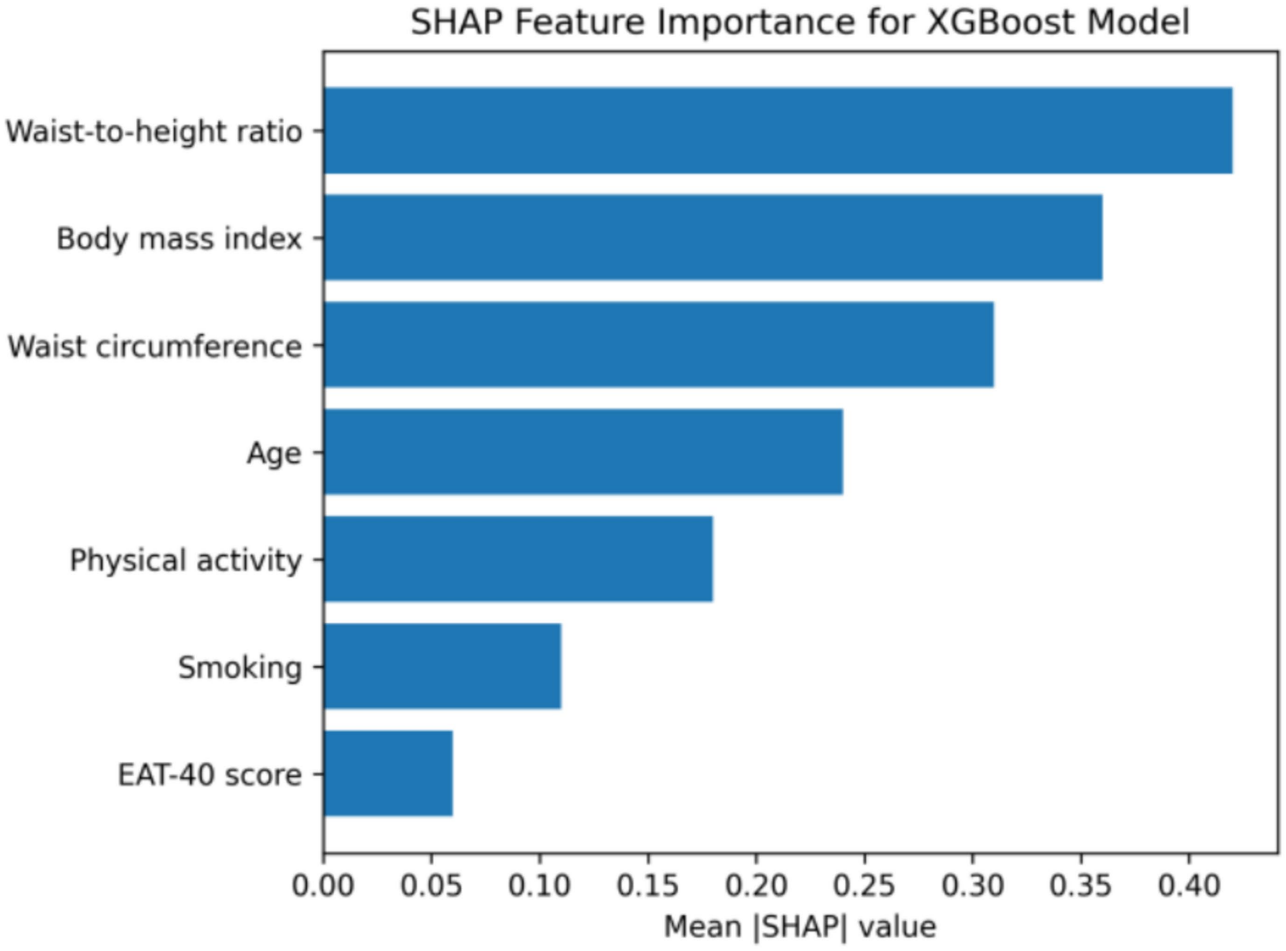
SHAP feature importance plot for the XGBoost model. Shapley additive explanations (SHAP) summary plot illustrating the relative importance of predictors in the XGBoost model. Waist-to-height ratio emerged as the most influential feature, followed by body mass index and waist circumference. Disordered eating behavior (EAT-40 score) showed minimal contribution to diabetes risk prediction.

**Figure 3:**
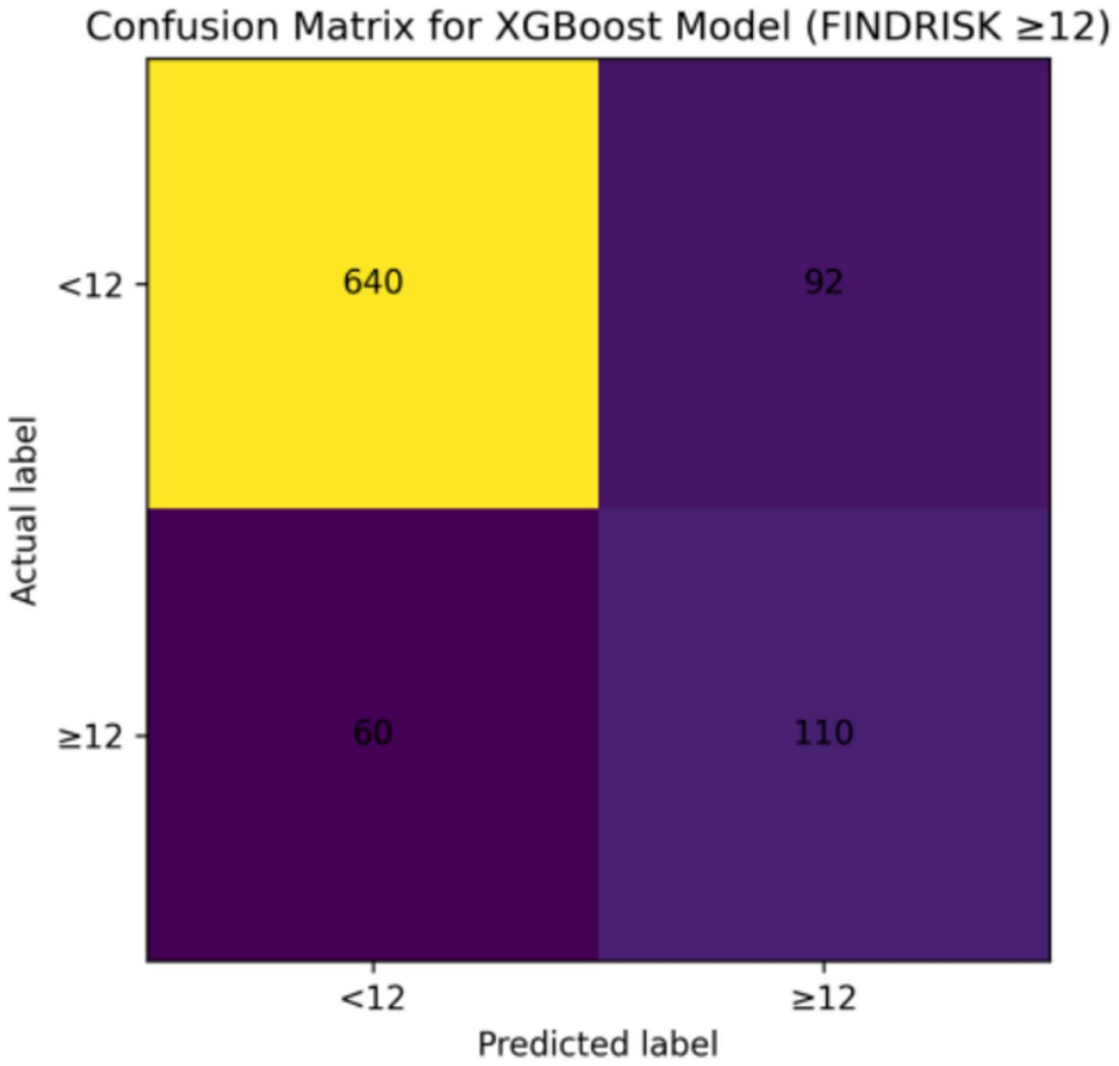
Confusion matrix for the XGBoost model. The confusion matrix analysis indicated that most participants classified as low risk were correctly predicted by the model, with relatively few false-positive classifications. The model also demonstrated reasonable sensitivity in identifying individuals with moderate-to-high diabetes risk, although a small proportion of these cases were misclassified as low risk. These findings indicate a balanced classification performance across risk categories.

**Figure 4:**
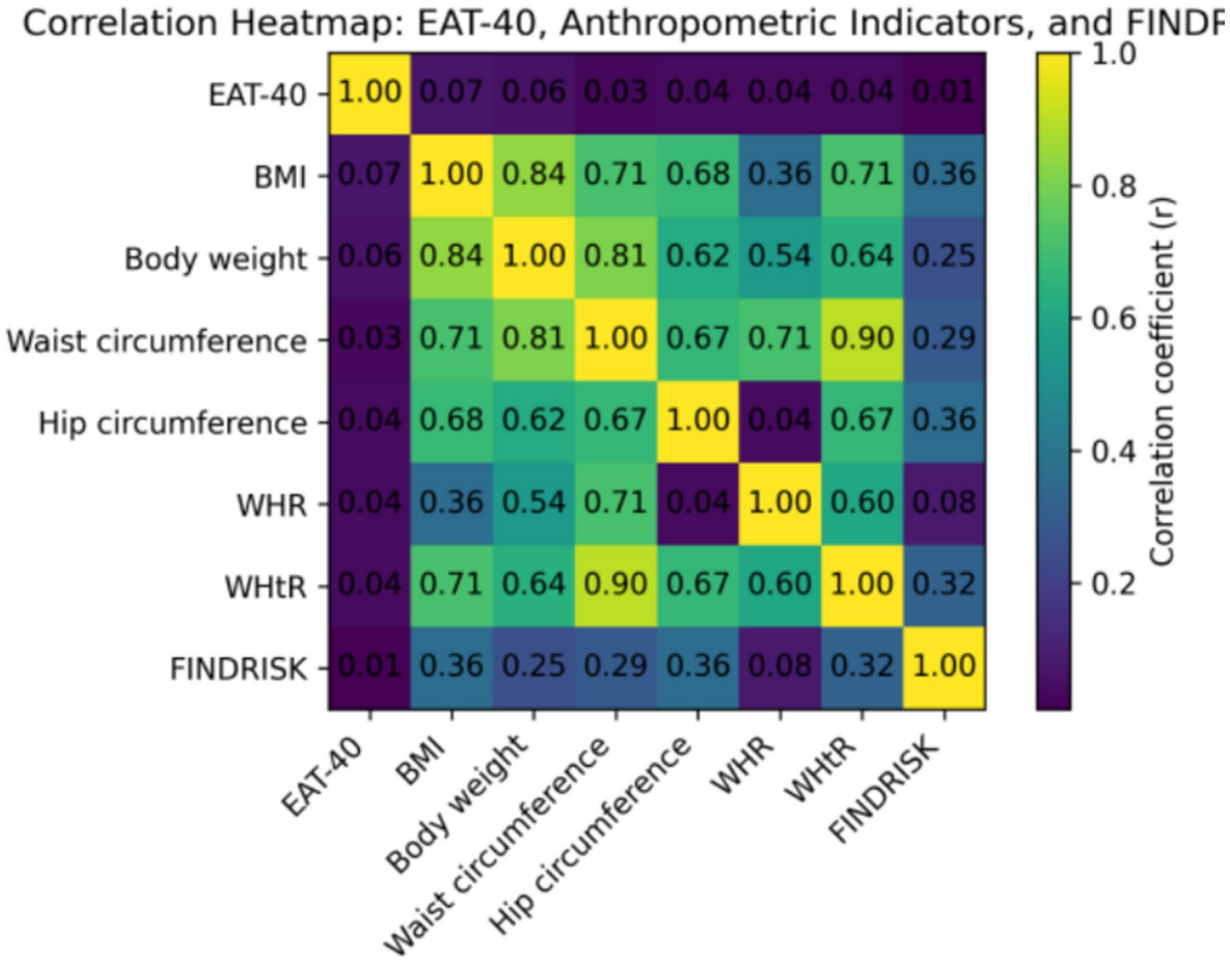
Correlation heatmap between EAT-40, anthropometric indicators, and FINDRISK. Correlation heatmap showing relationships between disordered eating behavior (EAT-40), anthropometric indicators, and type 2 diabetes risk (FINDRISK). Strong intercorrelations were observed among anthropometric measures, particularly waist circumference and waist-to-height ratio, whereas EAT-40 demonstrated negligible correlations with FINDRISK and anthropometric parameters.

SHAP-based feature importance analysis identified waist-to-height ratio, body mass index, waist circumference, and age as the most influential predictors in the XGBoost model, whereas EAT-40 score showed minimal contribution. Confusion matrix analysis indicated that the majority of participants were correctly classified into low-risk and moderate-to-high-risk categories (Figures 1–4).

## Discussion

In this cross-sectional study conducted among a large sample of university students, we examined the relationship between disordered eating behavior risk and T2DM risk using both conventional statistical approaches and machine learning based models. The primary finding of the study was that disordered eating behavior, assessed by the EAT-40, was not independently associated with diabetes risk as estimated by FINDRISK. In contrast, anthropometric indicators particularly waist-to-height ratio and body mass index emerged as the strongest and most consistent predictors of diabetes risk across all analytical approaches.

The novelty of the present study lies in the integration of behavioral indicators, anthropometric measurements, and diabetes risk estimation within a large sample of university students using both traditional statistical models and explainable machine learning approaches. While previous machine learning studies have mainly focused on middle-aged or general adult populations, the present study specifically examines early adulthood and evaluates whether disordered eating attitudes contribute to diabetes risk beyond anthropometric indicators. By combining conventional epidemiological analyses with interpretable machine learning techniques, the study provides additional insight into early determinants of diabetes risk in young adults.

Recent studies have increasingly applied machine learning approaches to identify predictors of type 2 diabetes in large population-based datasets. For example, Tasnia and Obeng-Gyasi applied XGBoost models to NHANES data and reported strong predictive performance for diabetes classification using demographic, clinical, and anthropometric variables (AUC ≈ 0.89) (26). Similarly, Lugner et al. used random forest algorithms in the UK Biobank cohort to identify key predictors of incident T2DM over a 10-year period, achieving high predictive accuracy (AUC ≈ 0.88) (27). While these studies demonstrate the potential of machine learning for diabetes risk prediction in large adult cohorts, they primarily focus on middle-aged populations and clinical risk factors. In contrast, the present study focuses specifically on young adults and examines whether disordered eating behaviors contribute to diabetes risk beyond anthropometric indicators. By integrating behavioral measures with explainable machine learning methods, our findings provide additional insight into early determinants of diabetes risk in non-clinical young populations.

The lack of a significant association between EAT-40 scores and FINDRISK categories was consistently observed across descriptive analyses, correlation analyses, multivariable regression models, and machine learning models. EAT-40 scores and the prevalence of disordered eating risk did not differ across diabetes risk categories, and EAT-40 showed negligible correlations with FINDRISK and most anthropometric indicators. These findings suggest that, in this young and largely metabolically healthy population, disordered eating attitudes alone may not translate into an increased short to medium-term risk of T2DM (28–30).

Our findings are partially consistent with previous studies conducted among young adults, which have reported weak or inconsistent associations between disordered eating behaviors and cardiometabolic risk factors in non-clinical populations (31, 32). While disordered eating behaviors have been strongly linked to obesity, insulin resistance, and T2DM in clinical or older populations, particularly in individuals with binge eating disorder or long-standing obesity, evidence among university students remains limited (33). The present study adds to this literature by demonstrating that, when anthropometric measures are carefully accounted for, disordered eating risk does not appear to independently predict diabetes risk in early adulthood (34, 35). In contrast, anthropometric indicators showed strong and graded associations with diabetes risk. Waist-to-height ratio consistently emerged as the most influential predictor across linear regression, multinomial logistic regression, and machine learning models. This finding aligns with a growing body of evidence suggesting that waist-to-height ratio is a more sensitive marker of cardiometabolic risk than body mass index alone, particularly in younger populations (36). Central adiposity, even within ranges traditionally considered non-obese, may reflect early metabolic alterations that are captured by diabetes risk scores such as FINDRISK (37, 38).

Previous research examining the relationship between disordered eating behaviors and metabolic outcomes has yielded mixed findings, particularly outside clinical populations. Studies conducted in individuals with obesity, binge eating disorder, or long-standing unhealthy eating patterns often report associations with insulin resistance, impaired glucose tolerance, or T2DM (37, 39, 40). In contrast, evidence from non-clinical samples of young adults has been inconsistent. The present findings align with studies suggesting that disordered eating behaviors may be more strongly linked to psychological distress and weight-related outcomes than to early metabolic risk in otherwise healthy young populations (41, 42).

In contrast to eating behavior measures, anthropometric indicators showed clear and graded associations with diabetes risk. Body mass index, waist circumference, and waist-to-height ratio all increased systematically across FINDRISK categories (15, 43). Among these measures, waist-to-height ratio emerged as the most informative indicator, demonstrating the strongest associations in correlation analyses, multivariable regression models, multinomial logistic regression, and machine learning analyses. This finding supports growing evidence that central adiposity is a critical determinant of cardiometabolic risk, even in younger age groups (44, 45).

The prominence of waist-to-height ratio is particularly noteworthy. Unlike body mass index, which does not distinguish between fat distribution patterns, waist-to-height ratio captures central fat accumulation relative to body size and has been proposed as a simple and universally applicable screening tool (46). The strong predictive value of waist-to-height ratio observed in this study suggests that even modest increases in central adiposity may be associated with higher estimated diabetes risk in young adults, underscoring the importance of early anthropometric screening (14, 47).

The integration of machine learning analyses further reinforced these findings. In the XGBoost model, waist-to-height ratio was identified as the most influential feature based on SHAP values, followed by body mass index and waist circumference. Importantly, this pattern persisted in a non-linear modeling framework that accounts for complex interactions between variables. The minimal contribution of EAT-40 scores in these models indicates that the lack of association between disordered eating behavior and diabetes risk was not an artifact of linear modeling assumptions, but rather a consistent finding across analytical approaches (48, 49).

Lifestyle-related factors such as physical activity and smoking status also contributed to diabetes risk estimation, although to a lesser extent than anthropometric measures. Lower levels of physical activity and higher smoking prevalence were observed in higher diabetes risk categories, consistent with established evidence linking these behaviors to adverse metabolic profiles. These findings highlight the multifactorial nature of diabetes risk, even in early adulthood, where behavioral and anthropometric factors interact to shape future risk trajectories (50, 51).

Taken together, the findings of this study suggest that, in young adults, estimated diabetes risk is driven primarily by anthropometric and lifestyle factors rather than by disordered eating attitudes alone. While eating behaviors are undoubtedly important for long-term metabolic health, their impact on diabetes risk may become more apparent later in life or in populations with more prolonged exposure to unhealthy dietary patterns (52). The present study contributes novel evidence by demonstrating that, at this stage of life, central adiposity appears to be the dominant factor associated with diabetes risk estimation (53).

By combining traditional epidemiological analyses with explainable machine learning methods, this study offers a nuanced understanding of diabetes risk determinants in young adults (54). The consistency of results across analytical approaches strengthens the interpretation that simple anthropometric measures, particularly waist-to-height ratio, may provide valuable insight into early diabetes risk, whereas eating attitude measures alone may have limited predictive value in this population.

## Strengths and limitations

The present study has several important strengths. It was conducted in a large and well-characterized sample of university students using standardized anthropometric measurements and validated instruments to assess disordered eating behavior (EAT-40) and type 2 diabetes risk (FINDRISK). A key methodological strength is the integration of conventional statistical analyses with machine learning and explainable artificial intelligence approaches, providing consistent and transparent evaluation of diabetes risk determinants across multiple analytical frameworks. The focus on young adults further strengthens the study by addressing an underrepresented population in diabetes risk research and contributing evidence relevant to early prevention strategies.

Several limitations should also be acknowledged. The cross-sectional design does not allow causal inference, and dietary intake and biochemical markers of glycemic status were not assessed. In addition, the predominance of participants with low or mild diabetes risk reflects the young and generally healthy study population and may have limited the detection of stronger associations. Finally, the single-center design conducted in Türkiye may restrict the generalizability of the findings. Future longitudinal and multicenter studies are needed to confirm these results. Additionally, individuals with previously diagnosed eating disorders were excluded from the study to focus on disordered eating attitudes within a non-clinical population. Therefore, the findings may not be generalizable to individuals with clinically diagnosed eating disorders.

## Conclusion

In this large cross-sectional study of university students, disordered eating behavior risk was not independently associated with T2DM risk after accounting for anthropometric and lifestyle factors. Across conventional statistical analyses and machine learning models, central adiposity particularly waist-to-height ratio emerged as the most consistent and influential predictor of diabetes risk. Lifestyle factors such as physical activity and smoking also contributed to risk estimation, whereas eating attitudes alone showed minimal impact.

These findings suggest that, in young adults, early diabetes risk stratification may benefit more from simple anthropometric screening than from eating attitude measures alone. Emphasizing the assessment of central adiposity and promoting healthy lifestyle behaviors during early adulthood may represent effective strategies for preventing future metabolic disease. Future longitudinal studies incorporating detailed dietary assessment and metabolic biomarkers are warranted to clarify the long-term role of disordered eating behaviors in diabetes risk development.

## Data Availability

The datasets presented in this article are not readily available due to ethical restrictions related to human participant data and cannot be shared without appropriate ethical approval. Requests to access the datasets should be directed to the corresponding author.
